# Investigation of Dendriplexes by Ion Mobility-Mass Spectrometry

**DOI:** 10.3390/molecules191220731

**Published:** 2014-12-12

**Authors:** Emma-Dune Leriche, Marie Hubert-Roux, Carlos Afonso, Catherine M. Lange, Martin C. Grossel, Florian Maire, Corinne Loutelier-Bourhis

**Affiliations:** 1 Normandie Université, COBRA, UMR6014 and FR3038, Université de Rouen; INSA de Rouen; CNRS, IRCOF, 1 rue Tesnière, 76821 Mont-Saint-Aignan Cedex, France; 2 School of Chemistry, University of Southampton, Highfield, Hants SO17 1BJ Southampton, UK

**Keywords:** polyamidoamine dendrimers, oligonucleotide duplex, dendriplexes, electrospray mass spectrometry, tandem mass spectrometry, ion mobility spectrometry

## Abstract

Highly branched polyamidoamine (PAMAM) dendrimers presenting biological activities have been envisaged as non-viral gene delivery vectors. They are known to associate with nucleic acid (DNA) in non-covalent complexes *via* electrostatic interactions. Although their transfection efficiency has been proved, PAMAMs present a significant cytotoxicity due to their cationic surface. To overcome such a drawback, different chemical modifications of the PAMAM surface have been reported such as the attachment of hydrophobic residues. In the present work, we studied the complexation of DNA duplexes with different low-generation PAMAM; ammonia-cored G0(N) and G1(N) PAMAM, native or chemically modified with aromatic residues, *i.e.*, phenyl-modified-PAMAM G0(N) and phenylalanine-modified-PAMAM G1(N). To investigate the interactions involved in the PAMAM/DNA complexes, also called dendriplexes, we used electrospray ionization (ESI) coupled to ion mobility spectrometry-mass-spectrometry (IM-MS). ESI is known to allow the study of non-covalent complexes in native conditions while IM-MS is a bidimensional separation technique particularly useful for the characterization of complex mixtures. IM-MS allows the separation of the expected complexes, possible additional non-specific complexes and the free ligands. Tandem mass spectrometry (MS/MS) was also used for the structural characterization. This work highlights the contribution of IM-MS and MS/MS for the study of small dendriplexes. The stoichiometries of the complexes and the equilibrium dissociation constants were determined. The [DNA/native PAMAM] and [DNA/modified-PAMAM] dendriplexes were compared.

## 1. Introduction

In the past decade, polyamidoamine (PAMAM) dendrimers have gained popularity in a variety of disciplines and particularly for their medical applications [1]. They present a well-defined structure with highly cationic surface that can be easily modified. Because of their chemical structures, PAMAM can develop attractive electrostatic interactions with nucleic acids and short oligonucleotides allowing the formation of complexes. It has been reported that complexes involving DNA and high generation PAMAM dendrimers, also called dendriplexes, were able to cross cell membranes, which is of great interest for their application in gene delivery [2]. PAMAMs do not present immunogenicity but the cationic surface of these compounds induces cytotoxicity which is however lower than the cytotoxicity of some polycationic polymers such as poly-L-lysine and polyethylenimine which are also under investigation as non-viral vector systems [3]. Chemical modifications of the dendrimers’ surface *via* the attachment of hydrophobic residues have been performed to reduce the toxic effects [4,5,6], although the hydrophobic residues must be carefully chosen. For instance, it was found that PEGylation and acetylation reduced the cytotoxicity profile, but the transfection ability of the PAMAM dendrimers was also significantly decreased [7]. It was shown that the surface modification of PAMAM dendrimers (generation G4) with aromatic amino acids greatly decreased the toxic effects and increased the transfection activity [8,9]. The modification may improve their ability to form complexes with DNA by adding hydrophobic or/and π stacking interactions to electrostatic interactions.

The studies of non-covalent complexes between DNA and high generation PAMAM have been usually performed using agarose gel electrophoresis [9], dynamic light scattering [8,10], fluorescence spectroscopy [10], circular dichroism [10], fluorescence microscopy imaging [10], and other techniques [11]. To our knowledge, ion mobility-mass spectrometry has never been used for the study of [DNA duplex/modified-PAMAM] dendriplexes. That could be explained by the very high mass of the [DNA/high generation PAMAM] complexes or by the complexity of some dendrimer samples (due to the presence of structural defects from PAMAM synthesis or degradation) that make difficult the MS analysis. However, it is well-known that mass spectrometry using the electrospray ionization technique under carefully controlled conditions can provide useful information concerning non-covalent complexes, such as oligonucleotide/drugs complexes. Determination of affinity, stoichiometry and equilibrium binding constants could be performed [12]. The combination with ion mobility allows an additional dimension of separation. IM is a post-ionization method used to separate gas phase ions according to their charge, their mass and their collision cross section (size and shape) [13]. The ions are accelerated by an electric field in a cell containing a buffer gas. In our case, the Travelling Wave Ion Mobility (TWIM) cell was used [14], N_2_ was the buffer gas and the electric field was non-uniform. The ions are slowed down by collisions with the buffer gas and separated when travelling the cell. The use of IM-MS has been particularly powerful for the separation of species with identical *m/z* ratios but different sizes (from different oligomeric orders and charge state for example) and/or different conformations [15]. Recently, IM-MS(/MS) has been successfully used to differentiate defective and ideal structures of low-generation PAMAM [16]; structural information as well as conformation study could be achieved. Otherwise, IM-MS has been an effective technique for the study of DNA structure [17,18] and complex mixtures of [DNA/ligand] complexes such as polyplexes [19,20].

In this work, we investigated the ability of low generation PAMAM, native or modified, to bind double-stranded DNA by ESI-IM-MS. We chose low generation PAMAM (G0 and G1) to form dendriplexes because these complexes can be analyzed by MS/MS under collision induced dissociation (CID) conditions that can provide information on the binding interactions, as previously reported for dendritic viologens/molecular tweezers complexes [21].

**Figure 1:**
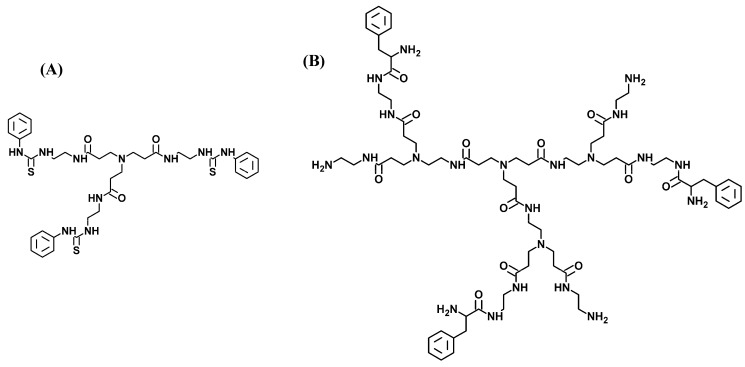
Chemical structures of phenyl-modified-PAMAM (generation 0) (**A**) and phenylalanine-modified-PAMAM (generation 1) [Phe_n_G1(N); n = 3] (**B**).

Note that they are very low generation dendrimers compared to dendrimers commonly used for dendriplex formation, so, they are more like ligands binding to the (much larger) DNA molecule than scaffolds that the DNA can wrap around/into, thus condensing the helix. However, these PAMAMs can constitute model systems to study the influence of surface chemical modifications with aromatic residues (phenyl group or phenylalanine) on their ability to form complexes with DNA. Thus, G0 phenyl-modified ammonia-cored PAMAM (φ_3_G0(N)), G1 phenylalanine-modified ammonia-cored PAMAM (Phe_n_G1(N); n = 0 to 3) (Figure 1) as well as the underivatized precursors were prepared and associated with the self-complementary (d(CGCGAATTCGCG)_2_) duplex which is a well-known system to investigate DNA binding drugs [22,23,24].

The resulting mixtures were analyzed by ESI-IM-MS. The stoichiometries of the complexes, the stabilities and the equilibrium dissociation constants were investigated. Tandem mass spectrometry experiments were also performed to study the dissociation behavior of the dendriplexes. Comparison of the different dendriplexes, [DNA duplex /native PAMAM] and [DNA duplex /modified-PAMAM] were discussed.

## 2. Results and Discussion

### 2.1. DNA Duplex

**Figure 2:**
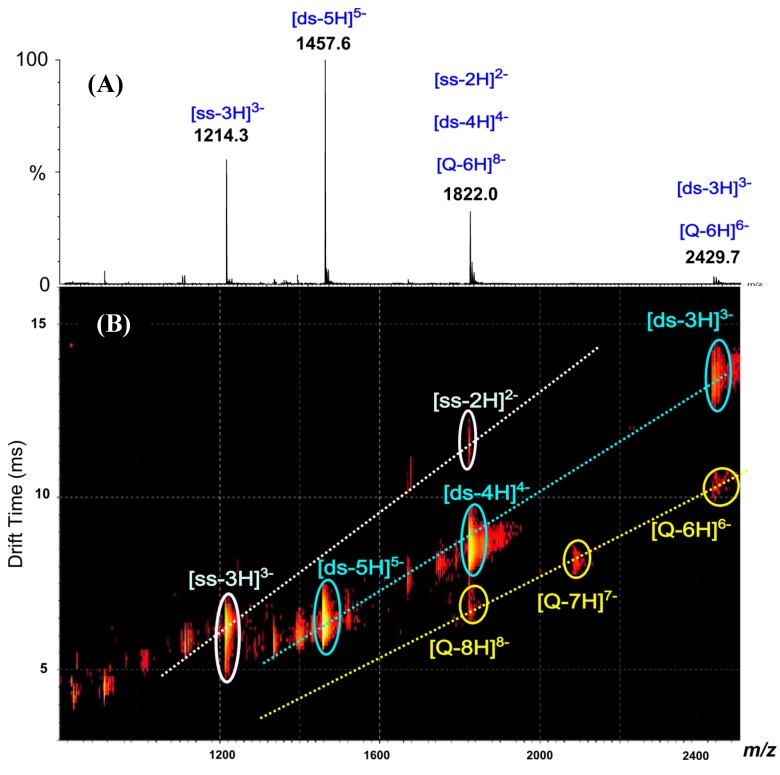
Negative-ion ESI mass spectrum (**A**) and IM-MS plot (drift time *versus m/z*) (**B**) of DNA duplex solution.

The DNA duplex was prepared and analyzed to optimize the sample preparation conditions and the instrumental conditions for the detection of non-covalent complexes. The sample of DNA duplex (*ds*) was prepared from the self-complementary d(GCGCAATTGCGC) single stranded oligonucleotide (*ss*) in 1 M ammonium acetate (pH = 6.7) using the annealing process described elsewhere [25]. After ultrafiltration, a solution of 17 µM DNA duplex in 0.1 M ammonium acetate (pH = 6.7) was directly analyzed by ESI-MS. Negative-ion detection mode was preferred due to the presence of numerous phosphate groups on DNA backbone. The ESI mass spectrum shows ions at *m/z* 1457.6, *m/z* 1822.0 and *m/z* 2429.7 which could correspond to [*ds*-5H]^5−^, [*ds*-4H]^4−^ and [*ds*-3H]^3−^ ions of DNA duplex, respectively (Figure 2A).

However, these ions could also be attributed to other species; for example the *m/z* 1822.0 could either correspond to [*ds*-4H]^4−^, to the doubly-charged ion of the single stranded oligonucleotide [*ss*-2H]^2−^ or to highly charged quadruplex ion [Q-8H]^8−^. In short, some single stranded DNA, duplex and quadruplex ions with even number of charges may have the same *m/z* ratio leading to overlapped ion signals. To unambiguously identify each signal, the separation of these species was achieved by ion mobility spectrometry. Indeed, the bidimensional drift time *vs. m/z* plot obtained from IM-MS analysis of the DNA duplex sample shows three diagonals which correspond to three ion series (Figure 2B): single stranded DNA ions ([*ss*-3H]^3−^ and [*ss*-2H]^2−^), duplex ions ([*ds*-5H]^5−^, [*ds*-4H]^4−^ and [*ds*-3H]^3−^) and quadruplex ions ([Q-8H]^8−^, [Q-7H]^7−^ and [Q-6H]^6−^). The IM-MS plot shows that the ions from DNA duplex constitute the major species of the sample (spots of higher intensities). Note that only the IM-MS coupling has permitted to unambiguously identify the ions of the duplex (these ions could be specifically extracted from the 2D-plot). Then, such results permitted to validate our solution of DNA duplex which has subsequently been used to study dendriplexes with either native or modified PAMAMs.

### 2.2. Dendriplexes 

The PAMAM G0(N) and G1(N) were synthesized according to Tomalia’s divergent approach [26] and were conjugated with phenyl groups or phenylalanine to yield φ_3_G(N) and Phe_n_G1(N) (*n* = 0 to 3), respectively. The chemical modification of G1(N) was performed using and adjusting previously reported methodology [27] and the grafting of phenylalanine residues yielded partially modified PAMAM (Phe_n_G1(N)) with *n* = 0, 1, 2 or 3. In other hand, the phenyl-modified-PAMAM (φ_3_G0(N)) was prepared by reaction of phenyl isothiocyanate on the PAMAM G0(N). The details of the synthesis and the characterization of all compounds are described in the Experimental Section.

Note that the phenyl-modified-PAMAM φ_3_G0(N) is a fully modified PAMAM which exhibits three grafted phenyl groups and no cationic surface, contrary to the native G0(N) PAMAM which presents three terminal amino groups (cationized at pH = 6.7). As the φ_3_G0(N) exhibits a highly hydrophobic surface, we expected that the [*ds*/φ_3_G0(N)] dendriplex could involve mainly hydrophobic and/or π stacking interactions while [*ds*/G0(N) PAMAM] dendriplex could involve mainly electrostatic interactions. We can also expect that G1(N) and Phe_n_G1(N) could present higher basicity than G0(N) because we can suppose that basicity should increase with the number of terminal amino groups. Consequently, we could expected a higher number of electrostatic interactions for G1(N) and Phe_n_G1(N) than for G0(N) and, all the more, for φ_3_G0(N).

To ensure that the experimental conditions and the instrumental parameters previously optimized for duplex analyses were also optimal conditions for DNA/ligand complexes, we carried out the analysis of a reference complex before infusing solutions of DNA duplex and PAMAM. The reference complex corresponded to a well-described system composed of equimolar mixtures of Dickerson duplex and of a model ligand, the diminazene, known to be a minor groove binder which exhibits high binding affinity with Dickerson duplex (data not shown). This way, we checked that DNA duplex/diminazene non covalent complex in 1:1 stoichiometry gave very abundant signals in accordance with previous data [25].

#### [ds/G0(N)] and [ds/φ_3_G0(N)] Dendriplexes

We mixed PAMAM with DNA duplex d(GCGCAATTGCGC)_2_ to yield solutions containing: (i) 17 µM of oligonucleotide and 17 µM of PAMAM in 0.1 M ammonium acetate (pH = 6.7), corresponding to 1:1 molar ratio (oligonucleotide:PAMAM) or (ii) 17 µM of oligonucleotide and 170 µM of PAMAM in 0.1 M ammonium acetate (pH = 6.7) corresponding to 1:10 molar ratio. Note that molar ratio description was preferred to charge ratio, commonly used to described dendriplexes because: (i) φ_3_G0(N) exhibits a lack of positive charge compared to other PAMAM compounds; (ii) mass spectrometric analyses of DNA/ligands are usually reported in molar ratio [12,23,25] and (iii) comparison between dendriplex and reference complex (DNA duplex/diminazene) can be made.

The negative-ion detection mode was also preferred for dendriplexes because of the presence of a high number of phosphate groups on the DNA backbone, higher than the number of positive charges that can hold PAMAM dendrimers which are very small compared to DNA duplex. Besides, it has been assumed that the ESI response factors of duplexes and DNA/ligand complexes were almost similar, according to previously reported data [12,23] where the DNA duplex and non-covalent complexes were supposed to present similar ESI response factors in the negative ESI ion mode, the ligand species being various basic drugs.

**Figure 3:**
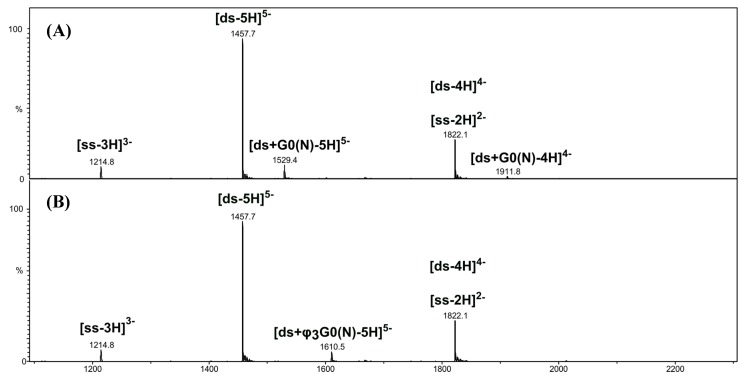
Negative-ion ESI mass spectra of [*ds*/G0(N)] (**A**) and [*ds*/φ_3_G0(N)] (**B**) dendriplexes solutions in 1:1 molar ratio.

The negative-ion ESI mass spectra of the [*ds*/G0(N)] and [*ds*/φ_3_G0(N)] dendriplexes in 1:1 molar ratio (17 µM of oligonucleotide and PAMAM) are displayed in Figure 3A,B, respectively. They both show abundant ions of the free DNA duplex ([*ds*-5H]^5−^, *m/z* 1457.7 and [*ds*-4H]^4−^, *m/z* 1822.1) as well as ions of single-stranded DNA, in lower abundance ([*ss*-3H]^3−^, *m/z* 1214.8 and ([*ss*-2H]^2−^, *m/z* 1822.1).

**Figure 4:**
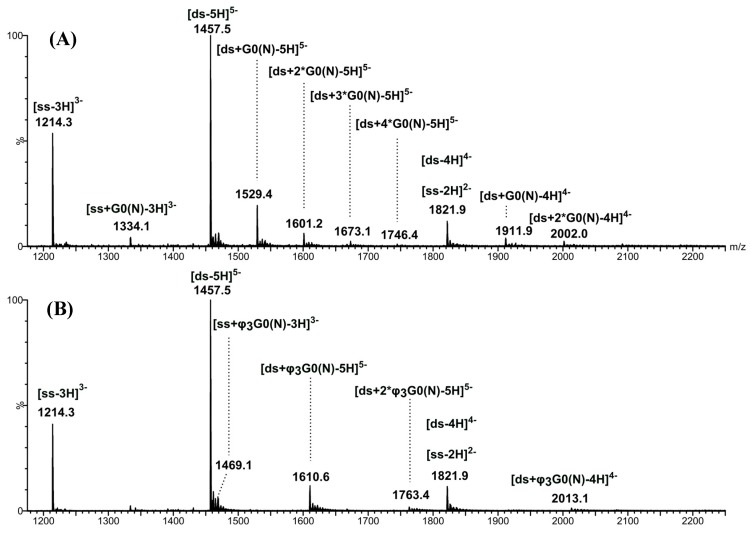
Negative-ion ESI mass spectra of [*ds*/G0(N)] dendriplex (**A**) and [*ds*/φ_3_G0(N)] dendriplex (**B**) in 1:10 molar ratio solution.

**Figure 5:**
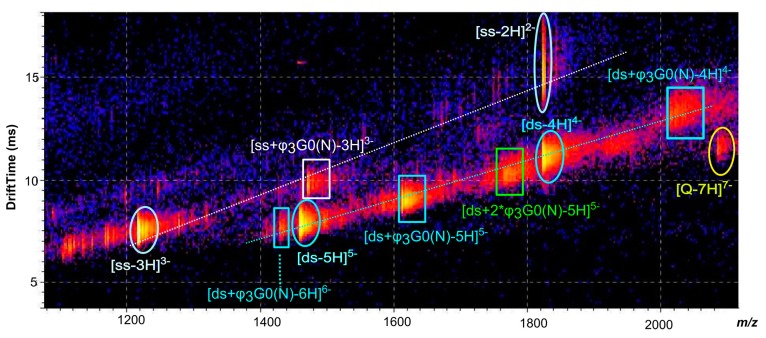
Negative-ion ESI IM-MS plot (drift time *vs.* *m/z*) of [*ds*/φ_3_G0(N)] dendriplex in 1:10 molar ratio solution.

In addition to DNA ions, other low-abundant ions were detected at *m/z* 1529.4 and 1610.5. They correspond to 5- charged ions of [*ds*/G0(N)] (Figure 3A) and [*ds*/φ_3_G0(N)] (Figure 3B) dendriplexes with 1/1 stoichiometry, respectively. A (4-) ion of [*ds*/G0(N)] dendriplex was also detected at *m/z* 1911.8, however in very low abundance (Figure 3A). The very low abundances of dendriplex ions compared to those of DNA duplex indicate a low binding affinity of G0(N) and φ_3_G0(N) for Dickerson duplex. However, no complex involving the single stranded DNA was observed, neither of [*ss*/G0(N)] nor [*ss*/φ_3_G0(N)], suggesting a preference of the non-covalent complexes for the double-stranded DNA. To assess the preference to form DNA duplex/PAMAM complexes, we analyzed the 1:10 molar ratio solution containing 17 µM DNA duplex and 170 µM PAMAM (Figure 4 and Figure 5).

When the dendrimers concentration increased, the intensities of the 5- charged ions of [*ds*/G0(N)] and [*ds*/φ_3_G0(N)] dendriplexes in 1/1 stoichiometry slightly increased and the 4- charged ions could now be detected for both dendriplexes (Figure 4A and Figure 4B). However, the free DNA duplex ions ([ds-5H]^5−^ , [ds-4H]^4−^ ) and the single stranded DNA ions ([ds-3H]^3−^ and [ds-2H]^2−^highlighted by IM-MS (Figure 5 in the case of [*ds*/φ_3_G0(N)] dendriplex) were still abundant, the most abundant signal corresponding to the [ds-5H]^5−^ ion (Figure 4). The main change in the ESI mass spectra of *ds*/PAMAM solutions in 1:10 molar ratio concerned the number of binding dendrimers that increased with the PAMAM concentration. Thus, 1/1 to 1/4 stoichiometries were observed for the 5- charged ion of [*ds*/G0(N)] dendriplex (*m/z* 1529.4, 1601.2, 1673.1 and 1746.4, respectively, in Figure 4A). In the case of [*ds*/φ_3_G0(N)] dendriplex, 1/1 to 1/2 stoichiometries were observed (*m/z* 1610.6 and 1763.4, respectively, in Figure 4B and Figure 5). The dendriplexes of higher stoichiometric ratio showed a decrease in intensities when the number of binding dendrimers increased. All these species as well as the free DNA duplex or the single strand are clearly highlighted in the bidimensional drift time *vs. m/z* plot obtained for the [*ds*/φ_3_G0(N)] dendriplex solution in the 1:10 molar ratio (Figure 5). Two diagonals are distinctly observed which correspond to two ion series: (i) free DNA duplex as well as [*ds*/φ_3_G0(N)] and [*ds*/(φ_3_G0(N))_2_] dendriplexes and (ii) free single stranded DNA and [*ss*/φ_3_G0(N)] complex. The non-covalent complexes between dendrimers and free single stranded DNA were observed for both G0(N) and φ_3_G0(N) PAMAM (*m/z* 1334.1 and *m/z* 1469.1 for [*ss*/G0(N)-3H]^3−^ and [*ss*/φ_3_G0(N)-3H]^3−^ dendriplexes, respectively) but in very low abundance. The 1/1 dendriplexes remained however the most abundant species (most numerous and most abundant signals). For example, the 1/1 [*ds*/φ_3_G0(N)] dendriplex predominated on [*ss*/φ_3_G0(N)] and [*ds*/(φ_3_G0(N))_2_] dendriplexes. The same trend was observed for The [*ds*/G0(N)] dendriplex.

Then, IM-MS experiment allows the detection and identification of some low-abundant ions such as [Q-7H]^7−^ ion or [*ss*/φ_3_G0(N)-3H]^3−^ ion which could not be readily observed in the mass spectrum because of their very low intensity and of possible overlapping with the DNA duplex ions .

Numerous non-covalent complexes are identified (*m/z* values in MS dimension) with more confidence due to the IM-MS dimension (*i.e*., diagonally aligned). Some of them could not be unambiguously detected by MS only.

Regarding the ion abundances of the 1/1 dendriplexes, the DNA duplex seems to bind both native G0(N) and modified φ_3_G0(N) PAMAMs with quite similar efficiencies, with a slight advantage for [*ds*/G0(N)] dendriplex. In the case of the native PAMAM, the binding may be explained by the possibility to form electrostatic interactions between the phosphate groups of the DNA duplex and the ammonium ions (charged terminal amino groups at pH = 6.7) of the native PAMAM. Hydrogen bonding interactions between the DNA bases and the PAMAM carbonyl groups as well as attraction interactions between the PAMAM ammonium groups and the π systems (electron rich π-system) of DNA backbone could not be excluded [28]. In the case of the φ_3_G0(N) ligand, hydrophobic, π stacking, hydrogen bonding interactions could be envisaged. A possible intercalation of phenyl end-groups of the modified-PAMAM between DNA bases at GC-rich regions could also be envisaged.

Then, that the presence of several stoichiometries (from 1/1 to 1/4 in the case of G0(N) ligand and 1/1 to 1/2 for φ_3_G0(N)) when PAMAM dendrimer concentration increased indicated that it was possible to attach several low generation PAMAM to DNA backbone or/and that some non-specific aggregation occurred. The maximum number of dendrimers that bound the DNA backbone is higher for G0(N) than for *ds*/φ_3_G0(N) (4 *vs.* 2). This phenomenon can be explained by electrostatic interactions between positively charged G0(N) PAMAM and DNA backbone while hydrophobic and/or π stacking interactions are more probably implied in [*ds*/φ_3_G0(N)] dendriplex formation, as previously mentioned.

Using ESI set in positive mode, the [*ds*/G0(N)] dendriplex ions were detected while [*ds*/φ_3_G0(N)] dendriplex ions were not observed (data not shown). The [*ds*/G0(N)] dendriplex was maintained in positive ion mode, because of the possibility of numerous electrostatic interactions. These interactions were less present in [*ds*/φ_3_G0(N)] dendriplex which was therefore weakened in positive ion mode. Moreover, it had been previously shown that, in the case of ammonium containing solutions analyzed in the ESI positive ion mode, the droplet surface excess charges are ammonium and the extra positive surface charge can lead to the disturbance or disruption of [DNA/ligand] complexes, all the more the weakest ones [29]. Gabelica *et al.* reported that abundant 1/1 and weak 1/2 DNA duplex/drug complexes could be observed for intercalators in ESI negative ion mode and that sometimes no complex could be observed in the positive ion mode for some particular intercalators that interact by the sole staking interaction mode [29]. Thus, intercalation for [ds/φ_3_G0(N)] dendriplex formation can be envisaged because of the presence of the planar phenyl groups which can bind to DNA by insertion of these aromatic moieties between a DNA base pairs.

The equilibrium dissociation constants (*K*_D_) of 1/1 complexes were determined in the negative-ion mode by ESI-MS experiments for dendriplex solutions in 1:1 molar ratio, using a well-described methodology in ESI-MS [12]. Some measurements were also carried out by ESI-IM-MS for comparison. The possibilities to determine equilibrium constants by ESI-MS have been widely reported [23,28,29,30,31] and ESI-MS proved to be a reliable technique for stoichiometries, relative binding affinities and equilibrium constants determination. The main difficulties lay in: (i) replacement of physiological buffers by ammonium acetate more compatible with ESI ionization but which can induce discrepancies and (ii) estimation of response factors of the duplex and complexes, *i.e.* dendriplexes in our specific case. To determine the equilibrium dissociation constants of the dendriplexes, we measured the signal of the ion of the free DNA duplex and the ion of the dendriplex (1/1 stoichiometry) detected in the equimolar solution at 17 μM. These estimations were performed according to [12] assuming that DNA duplex and dendriplex ions have similar response factors, as previously mentioned. To validate our results, we also determined the *K*_D_ of the reference DNA duplex/diminazene complex and verified that our estimation was consistent with the literature. Note that the integration of an ion can be more accurate by IM-MS than by MS alone since we can specifically extract the dendriplex and duplex signals from the 2D plot before integration (the background can be removed and the signal-to-noise ratio improved by elimination of contaminant signals).

The *K*_D_ values were estimated to be nearly 2 × 10^−4^ M for [*ds*/G0(N)] and 3 × 10^−4^ M for [*ds*/φ_3_G0(N)] dendriplexes. The *K_D_*s of dendriplexes were also determined by competition experiments. A solution containing the DNA duplex, φ_3_G0(N) and G0(N) in 1:0.5:0.5 molar ratio was analyzed by ESI-MS. The [*ds*/G0(N)] and [*ds*/φ_3_G0(N)] dendriplexes were detected and their *K_D_* were both around 5–6 × 10^−4^ M.

The *K*_D_ values obtained for both [*ds*/G0(N)] and [*ds*/φ_3_G0(N)] dendriplexes show that after chemical modification, the affinity of the dendrimer for the DNA duplex does not significantly change even if the interactions involved in the dendriplex formation could be from different nature (distinct behaviors of [*ds*/G0(N)/] and [*ds*/φ_3_G0(N)] dendriplexes in the positive-ion mode or when the concentration of dendrimers increased suggesting that the non-covalent interactions involved in the two dendriplexes could differ).

To get more insight into the interactions involved in the [*ds*/PAMAM] dendriplexes formation, CID experiments were performed and MS/MS spectra selecting the 5- charged ion as precursor. We expected that the dissociation study of dendriplexes with 1/1 stoichiometry could afford information concerning the reactivity and stability of the non-covalent complexes. MS/MS experiment selecting the 5- charged DNA duplex ion ([ds-5H]^5−^) was also performed for comparison.

**Figure 6:**
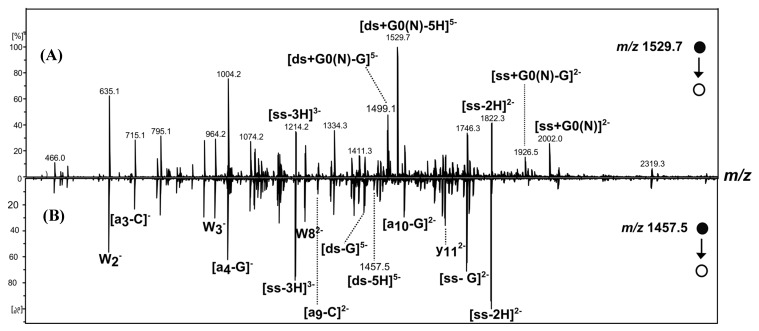
Negative-ion ESI MS/MS spectra of [*ds*/G0(N)-5H]^5−^ (*m/z* 1529.7) (**A**) and [*ds*-5H]^5−^ (*m/z* 1457.5) (**B**).

**Figure 7:**
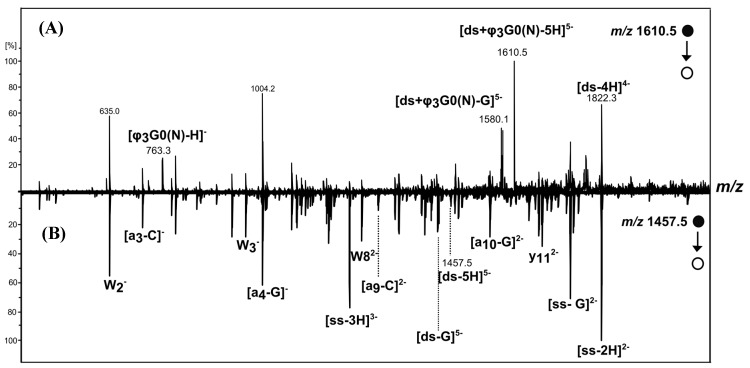
Negative-ion ESI-MS/MS spectra of [ds/φ_3_G0(N)-5H]^5−^ (*m/z* 1610.5) (**A**) and [ds-5H]^5−^ (*m/z* 1,457.5) (**B**).

Negative-ion ESI-MS/MS spectra of [(*ds*/G0(N))-5H]^5−^(*m/z* 1,529.7), [*ds*/φ_3_G0(N)-5H]^5−^(*m/z* 1610.5) and [ds-5H]^5−^(*m/z* 1457.5) show, in addition to the precursor ion, several product ions (Figure 6 and Figure 7). Some of the product ions were characteristic of the DNA structure such as a-C, a-G, w, y, ds-G and ss-G type-ions (oligonucleotide product ions nomenclature according to McLuckey *et al.* [32]). They were observed for both dendriplexes and for the DNA duplex (comparison with the MS/MS spectrum of [ds-5H]^5−^ displays in Figure 6B and Figure 7B). Other product ions are characteristic of the dendriplexes:

- Some arise from common fragmentation pathway involving the neutral loss of guanine from the 5- charged precursor; for example the ions at *m/z* 1,499.1 and *m/z* 1,580.1 which correspond to [*ds*/G0(N)-G]^5−^ and [*ds*/φ_3_G0(N)-G]^5−^, respectively.

- Others arise from different dissociation pathways. Thus, dissociation pathways of [*ds*/G0(N)-5H]^5−^ involve the separation of the strands leading to product ions such as [*ss*/G0(N)-2H]^2−^ (*m/z* 2002.0), [*ss*/ G0(N)-2H-G]^2−^ (*m/z* 1926.5), [ss-2H]^2−^ (*m/z* 1822.3) and [*ss*-3H]^3−^ (*m/z* 1214.2) involving single stranded DNA, in complex or free. This pathway dissociation is consistent with the assumption that electrostatic interactions are involved in the formation of the non-covalent complexes between the native PAMAM and the DNA duplex.

On the other hand, the 5- charged ion [*ds*/φ_3_G0(N)-5H]^5−^ dissociates into the [φ_3_G0(N)-H]^−^ (*m/z* 763.3) and [*ds*-4H]^4−^ (*m/z* 1822.3) product ions which correspond to the modified PAMAM and to the DNA duplex moieties, respectively (Figure 7). The detection of these two complementary product ions and the absence of [*ss*/φ_3_G0(N)-nH]^n−^ or [*ss*/φ_3_G0(N)-nH-G]^n−^ type-ions supports the assumption that the non-covalent complexes between φ_3_G0(N) and the DNA duplex may be formed by intercalation, according to Gabelica *et al.* [29] with insertion of the phenyl group of φ_3_G0(N) between DNA base pairs.

### 2.3. [ds/G1(N)/] and [ds/Phe_n_G1(N)] Dendriplexes

The larger synthetized native PAMAM G1(N) as well as the partially modified PAMAM G1(N) with phenylalanine were also investigated. The dendriplex solutions were submitted to both approaches, ESI-QIT-MS(/MS) and ESI-Q-IM-TOF-MS. 

The negative-ion ESI mass spectrum of the [*ds*/G1(N)] dendriplex solution in 1:1 molar ratio shows the 5- charged ion of [*ds*/G1(N)] dendriplex with 1/1 stoichiometry (data not shown), as previously observed in the case of G0(N) (Figure 3A). For the equimolar dendriplex solutions, PAMAM G0(N) and PAMAM G1(N) present a similar behavior regarding the interaction with the DNA duplex. The *K_D_* of [*ds*/G1(N)] dendriplex with 1/1 stoichiometry was estimated to be about 2 × 10^−4^ M, in the same range of values as the *K_D_* of [*ds*/G0(N)] or of [*ds*/φ_3_G0(N)] dendriplexes. Besides, the mass spectrum of [*ds*/G1(N)] dendriplex solution in 1:10 molar ratio shows dendriplexes with 1/1 and 1/2 stoichiometries at *m/z* 1666.2 and 1875.2, respectively, while the 1/1 to 1/4 stoichiometries were previously observed in the case of the [*ds*/G0(N)] dendriplex solutions in 1:10 molar ratio. This difference can be explained by the fact that G1(N) is much larger than G0(N) and do not aggregate as much as G0(N). The ESI mass spectrum of the [*ds*/G1(N)] dendriplex solution also depicts the 3- charged ion of [*ss*/G1(N)] complex, lower than the 5- charged ion of [*ds*/G1(N)] dendriplex. The presence of such ion was not expected; some explanations can be suggested, such as a decomposition of DNA duplex during the sample preparation of infusion leading to non-specific aggregation or in-source dissociation of the 5- charged ion of [*ds*/G1(N)] dendriplex.

**Figure 8:**
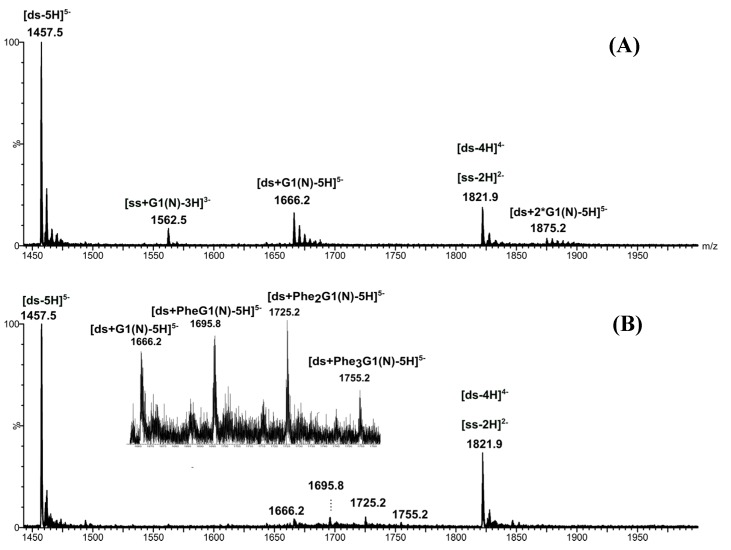
Negative-ion ESI mass spectra of [*ds*/G1(N)] (**A**) and [*ds*/Phe_n_G1(N)] (**B**) dendriplexes in 1:10 molar ratio solution. Insert: enlargement of *m/z* 1660–1763 range (dendriplexes ions region).

The ESI mass spectra of the [*ds*/Phe_n_G1(N)] dendriplex solutions in either 1:1 or 1:10 molar ratios show the 5- charged ions of the dendriplexes [*ds*/G1(N)], [*ds*/PheG1(N)], [*ds*/Phe_2_G1(N)] and [*ds*/Phe_3_G1(N)] with 1/1 stoichiometry (Figure 8B with 1:10 molar ratio). These ions were detected at *m/z* 1666.2, *m/z* 1695.8, *m/z* 1725.2 and *m/z* 1755.2, respectively, but with very low intensities.

**Figure 9:**
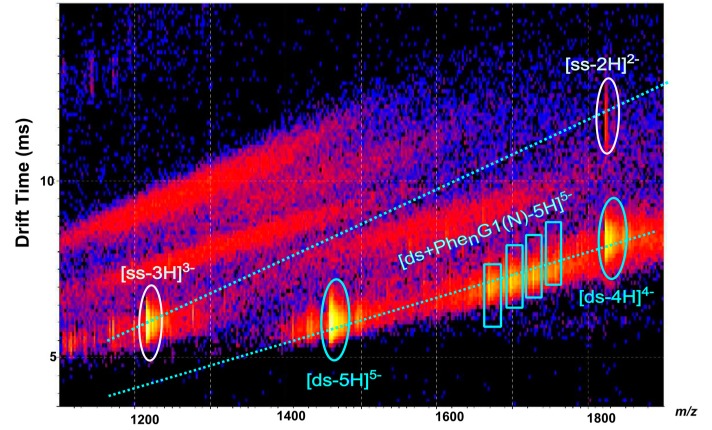
Negative-ion ESI IM-MS plot (drift time *vs. m/z*) of [*ds*/Phe_n_G1(N)] dendriplex in 1:10 molar ratio solution.

The distribution of these [*ds*/Phe_n_G1(N)] dendriplex ions was more evidenced using IM-MS; the 5- charged ions were observed on the same diagonal and were almost aligned with the 5- charged ions of the DNA duplex (Figure 9). The 2D ESI-IM-MS plot also depicts single stranded DNA ions ([ss-3H]^3−^ and [ss-2H]^2−^), as previously observed in Figure 2 and Figure 5. No [*ss*/modified-dendrimer] complex ion was observed. Independently of the concentration of dendrimer used in this study, the modified Phe_n_G1(N) PAMAM interact only with the DNA duplex to yield a 1/1 stoichiometry dendriplex. A linear correlation was observed between the drift time and the number of grafted phenylalanines; as expected, the drift time increased with the number of grafted phenylalanines because the size of the modified dendrimer gradually increases at each grafting stage.

The dissociation constants were determined for each dendriplexes (keeping in mind that signal-to-noise ratio remain low for the dendriplex ions) and were in the range of 10^−3^ M, which is the upper limit for *K_D_* determination by mass spectrometry (*K_D_* from 10^−3^ to 10^−8^ M can be determined by ESI-MS [12]). The [*ds*/Phe_3_G1(N)] dendriplex showed the highest *K_D_* value. These high *K_D_* values can be explained by the competition between these modified-dendrimers to interact with the DNA duplex. However, the affinities of native or phenylalanine-modified G1(N) PAMAM for the DNA duplex are still very weak meaning that either the formation of [*ds*/Phe_n_G1(N)] dendriplexes is not favored, the approximation of similar response factors between duplex and dendriplexes is no longer valid or that some chemical species present in the sample would prevent the dendriplex formation. This disturbance could be due to a reagent used during the dendrimer synthesis which is not totally removed after the purification steps.

## 3. Experimental Section

### 3.1. Reagents

*N*-(*tert-*Butoxycarbonyl)-phenylalanine (Boc-Phe-OH), phenyl isothiocyanate, *O*-(benzotriazol-1-yl)-*N,N,N′,N′*-tetramethyluronium hexafluorophosphate (HBTU), 1-hydroxybenzotriazole hydrate (HOBt hydrate), *N,N*-dimethylformamide (DMF), *N,N*-diisopropylethylamine (DIPEA), phosphoric acid (H_3_PO_4_), dichloromethane (DMC), HPLC grade methanol and oligonucleotide d(CGCGAATTCGCG) were purchased from Sigma-Aldrich (Saint-Quentin-Fallavier, France). 2,2,2-Trifluoroethanol (TFE), phosphoric acid (H_3_PO_4_), Float-A-Lyzer G2 dialyzer (1 kDa), ammonium acetate and LC-MS grade acetonitrile (ACN) were purchased from Fisher Scientific (Illkirch, France). Deionised water (18MΩ) was obtained from a Milli-Q apparatus (Millipore, Bedford, MA, USA). Amicon Ultra 0.5 mL centrifugal filters (MWCO 3000) were purchased from Millipore (Molsheim, France).

### 3.2. Synthesis and Chemical Modification of PAMAM

The PAMAM G0(N) and G1(N) were synthesized according to Tomalia’s divergent approach [26].

*PAMAM G0 Ammonia Core [G0(N)]:* A solution of methyl acrylate (2.14 equiv per dendrimer amine end group, 176 mL, 1.94 mol) in methanol (50 mL) was added dropwise over six hours to a stirred solution of ammonia (5.85 mL, 0.305 mol) in methanol (50 mL). The reaction mixture was stirred at room temperature in the dark for three days then concentrated *in vacuo.* An azeotropic distillation was performed as follows: toluene (30 mL) was added to the product dissolved in methanol (3 mL). The solvents were then removed *in vacuo*. This was repeated a further two times. The product was then dissolved in methanol (10 mL), which was then removed *in vacuo* to give the half-generation PAMAM G-0.5 (117%; product contaminated with solvent). ^1^H-NMR (300 MHz, CDCl_3_): δ = 3.60 (9H, s, H-6); 2.70 (6H, t, H-2); 2.42 (6H, t, H-3). ^13^C-NMR (75 MHz, CDCl_3_): δ = 172.9 (C-4); 51.9 (C-6); 49.2 (C-2); 32.9 (C-3).

To a solution of ethylenediamine (EDA) (10 equiv. per dendrimer ester end group; 108 mL, 1.61 mol) in methanol (50 mL) was directly added a solution of half-generation PAMAM G-0.5 (15 g, 54.54 mmol) in methanol (25 mL) then stirred for six days at room temperature. The full generation PAMAM G0 was obtained after a repetition azeotropic distillation [toluene (100 mL) was added to the product dissolved in methanol (10 mL)] (yield: 110%; product contaminated with solvent). ^1^H-NMR (300 MHz, D_2_O): δ = 3.22 (6H, t, H-6); 2.79 (6H, t, H-2); 2.73 (6H, t, H-7); 2.40 (6H, t, H-3). ^13^C-NMR (75 MHz, D_2_O): δ = 175.7 (C-4); 49.6 (C-2); 42.7 (C-6); 41.8 (C-7); 33.9 (C-3).

*Phenyl-Modified PAMAM G0 Ammonia Core [φ_3_G0(N)]*: The phenyl-modified-PAMAM (φ_3_G0(N)) was prepared by reaction of phenyl isothiocyanate on the PAMAM G0(N). Thus, to a solution of phenyl isothiocyanate (4.5 equiv*.*), in DMF (5 mL) was added dropwise a solution containing PAMAM G0(N) (1 equiv.) and triethylamine (374 μL) in H_2_O (2 mL). The whole mixture was stirred for three days at room temperature. The solvent was removed and the compound was purified by column chromatography (Rf: 0.26, 10% MeOH in DCM, silica 60) to give phenyl-modified-PAMAM G0 ammonia-cored [φ_3_-G0(N)] (yield: 80%). ESI-HRMS: *m/z* calculated and measured for C_36_H_48_N_10_N_3_S_3_ ([M+H]^+^) were, 765.3151 and 765,3148, respectively. ^1^H-NMR (600 MHz, CD_3_OD): δ = 9.69, 8.05, 7.84 (br.s, NH), 7.41 (6H, d, H-12), 7.31 (6H, t, H-13), 7.09 (3H, t, H-14), 3.54 (6H, s (br.d), H-7), 3.25 (6H, s (br.d), H-6), 2.67 (6H, (br.s), H-2), 2.23 (6H, (br.s), H-3); ^13^C-NMR (150 MHz, CD_3_OD): δ = 180.6 (C, C-9), 169.1 (C, C-4), 128.5 (CH, C-12), 124.1 (CH, C-13), 123.2 (CH, C-14), 48.9(CH_2_, C-2), 43.2 (CH_2_, C-6 and C-7), 38.06 (CH_2_, C-3). (Carbon labelling is reported in the supplementary information; Figure S1A).

*PAMAM G1 Ammonia Core [G1(N)]*: A solution of methyl acrylate (131 mL, 1.45 mol) in methanol (50 mL) was added dropwise to a stirred solution of G0 (14.50 g, 40.38 mmol) in methanol (60 mL). The reaction mixture was stirred at 30 °C. After one hour the temperature was allowed to reach ambient, the whole mixture was stirred for three days in the dark then concentrated *in vacuo* followed by a repetitive azeotropic distillation [toluene (100 mL) was added to the product dissolved in methanol (10 mL)]. The crude product was purified by column chromatography (Rf: 0.40, 10% MeOH in DCM, silica 60) to give half-generation PAMAM G0.5 (yield: 53%). ^1^H-NMR (300 MHz, D_2_O): δ = 7.05 (18H, s, NH); 3.66 (18H, s, H-13); 3.20 (6H, q, H-6) 2.70 (18H, m, H-2 and H-9); 2.50 (6H, t, H-7); 2.40 (6H, t, H-3 and H-10). ^13^C-NMR (75 MHz, D_2_O): δ = 173.0 (C-11); 172.1 (C-4); 53.1 (C-2); 51.6 (C-13); 51.5 (C-7); 49.3 (C-9); 37 (C-6); 33.6 (C-3); 32.7(C-10). (Carbon labelling reported in Figure S1B).

To a solution of ethylenediamine (EDA) (1.60 mol) in methanol (32 mL) was directly added a solution of half-generation PAMAM G0.5 (4.70 mmol) in methanol (13 mL) then stirred for five days at room temperature. An azeotropic distillation was performed [toluene (30 mL) was added to the product dissolved in methanol (3 mL)] giving the full generation PAMAM G1 (yield: 142%; product contaminated with EDA). ESI-HRMS: m/z calculated and measured for C_45_H_93_N_19_O_9_ ([M + H]^+^) were 1044.7482 and: 1044.7487, respectively. ^1^H-NMR (300 MHz, D2O): δ =3.26 (6H, t, H-6), 3.21 (12H, t, H-13’), 2.79 (12H, t, H-14’), 2.76 (6H, t, H-2), 2.69 (12H, t, H9’), 2.60 (6H, t, H7), 2.40 (18H, m, H-3 and H10’).

*Phenylalanine-Modified PAMAM G1 Ammonia Core [PhenG1(N)]:* The chemical modification of G1(N) was performed according to previous data [27]. The grafting of phenylalanine residues yielded partially modified PAMAM (Phe_n_G1(N)) with n = 0, 1, 2 or 3.

A solution containing 3 equiv. of *N-Boc*-Phe-OH, 3.3 equiv. of HBTU, HOBt•H_2_O and DIPEA in DMF (5 mL) was prepared at 0 °C and stirred for 40 minutes. To this solution was added dropwise a solution of PAMAM G1(N) (0.95 equiv.) in DMF/water (5:2 *v*/*v*). The whole was then allowed to stir at 0 °C for two hours, then at room temperature for a night. The product was dialyzed (*Mw* cutoff = 1000 Da) for 3 days against deionized water; the dialysed sample was then lyophilized. The N-Boc protecting group was removed in TFE by adding 17 equiv. of aqueous H_3_PO_4_ (85% Wt). The reaction mixture was stirred at room temperature in dark for 12 min (a precipitate was formed almost instantaneously). The reaction mixture was then diluted in H_2_O and the pH was adjusted to ≈ 7 with ammonium acetate (1 M). The product was dialyzed for 6 days against deionized water and lyophilized to give a bruin solid (yield 51%). Elemental composition of the different Phe_n_G1(N) were determined by accurate mass measurements (Table 1) according to previous method [27].

**Table 1:** ESI-HRMS: Elemental compositions of PhenG1(N) modified PAMAM (n = 0 to 3) determined by accurate mass measurements.

Compound	Ion	*m*/*z* Exp. ^a^	*m*/*z* Calc. ^b^	Error (ppm)	Elemental Compositions ^c^
G1(N)	[M+2H]^2+^	522.8770	522.8780	1.90	C_45_H_93_N_19_O_9_
PheG1(N)	[M+2H]^2+^	596.4121	596.4122	−0.08	C_54_H_102_N_20_O_10_
Phe_2_G1(N)	[M+2H]^2+^	669.9465	669.9464	0.10	C_63_H_111_N_21_O_11_
Phe_3_G1(N)	[M+2H]^2+^	743.4806	743.4807	−0.06	C_72_H_120_N_22_O_12_

^a^ Accurate measurements performed using a Waters SYNAPT G2 hybrid quadrupole/HDMS instrument equipped with an ESI LockSpray™ source and operated in “V” resolution mode (resolution 20000 FWHM). Leucine enkephalin (2 ng/µL) was used as the lock mass and was infused (10 µL/min) using an independent reference spray via the LockSprayTM interface which was operated at a reference scan frequency, lock spray capillary and collision energy of 10s, 3 kV and 4 eV, respectively. ^b^ Monoisotopic values calculated with the Waters MassLynx software. ^c^ Elemental compositions of the neutral species.

^1^H-NMR (600 MHz, D_2_O): δ = 7.10–6.97 (5H, br.s, H-21, 22 and 23), 3.57 (1H, br.s, H-17), 3.21 (10H, br.s, H-13’), 3.05 (10H, m, H-6, 13 and 14), 2.85 (10H, br.s, H-14’), 2.73 (2H, t, H-19), 2.57 (18H, m, H-2, 9 and 9’), 2.39 (6 H, m, H-7), 2.21 (12H, t, H-10 and 10’), 2.17 (6 H, br.s, H-3); ^13^C-NMR (150 MHz, D_2_O): δ = 175.0 (C, C-4, 11 and 16), 50.4 (CH_2_, C-7), 48.5 (CH_2_, C-2 and 9), 39.0 (CH_2_, C-14’ and 19), 38.8 (CH_2_, C-13), 36.5 (CH_2_, C-13’), 36.0 (CH_2_, C-14), 32.0 (CH_2_, C-3, C-10 and C-10’). (Carbon labelling reported in Figure S1C).

The CID mass spectra (ESI-MS/MS) of all compounds were registered and showed the characteristic fragmentation pattern of PAMAM compounds including successive native and/or modified generational branch and/or arm losses. Such results were consistent with and our previous work [27,33,34]. More specific decomposition of protonated molecule of φ_3_G0(N) is reported in the supplementary information (Figure S2). The ESI-MS/MS spectrum and the fragmentation pattern of [Phe_2_G1(N)+2H]^2+^ is reported in Figure S3.

### 3.3. Preparation of Samples 

*DNA duplex (d(CGCGAATTCGCG)_2_)*: A solution of 60 μL of 0.8 mM self-complementary oligonucleotide d(CGCGAATTCGCG) in 1 M ammonium acetate were annealed by heating to 85 °C for 10 min and cooling to room temperature over three hours. Then, the duplex solution (50 μL) was diluted in 100 mM ammonium acetate (400 μL). The resulting solution was then purified and concentrated (ultrafiltration; 2 × 10 min) using amicon Ultra 0.5 mL centrifugal Filters (MWCO 3000). Then, the sample was diluted in a mixture of ammonium acetate and methanol which was added to ensure the stability of the spray; the final solution we analyzed contained 17 μM of duplex solution in (20/80: methanol/100 mM ammonium acetate, pH = 6.7) solvent system. 20% methanol is the upper limit admissible that allows a stable spray without disturbing the complex stability [12].

*[ds/Dendrimer] dendriplexes*: 10 μL of solution containing the duplex (0.4 mM) was interacted with either 10 μL of 0.4 mM (1:1 molar ratio) or 10 μL of 4 mM (1:10 molar ratio) dendrimers (G0(N), G1(N), φ_3_G0(N) and Phe_n_G1(N)). The mixtures were then diluted in (20/80: methanol/100 mM ammonium acetate); the 1:1 final solutions contained 17 μM of duplex and 17 μM of dendrimer in (20/80: methanol/100 mM ammonium acetate, pH = 6.7); thus, the 1:10 final solutions contained 17 μM of duplex and 170 μM of dendrimer in (20/80: methanol/ 100 mM ammonium acetate, pH = 6.7). All the final solutions were directly infused into the ESI source and analyzed without further purification.

### 3.4. ESI-Q-TOF-MS(/MS) and ESI-IM-MS of Dendriplexes [ds/Native PAMAM] and [ds/Modified-PAMAM]

*ESI-Q-(IM)-MS(/MS)*: These experiments were performed using a Waters SYNAPT G2 hybrid quadrupole/time of flight (Q/TOF) HDMS instrument equipped with an ESI LockSpray™ source, the MassLynx 4.1 and the DriftScope 2.2 softwares (Waters, Manchester, UK). The hybrid geometry of the MS system and the IMS “Triwave” module consisting of three travelling wave-enabled stacked ring ion guides that have been described elsewhere [14,35]. The SYNAPT HDMS system was calibrated using sodium formate cluster ions (2 mg·mL^−1^) and operated in “V” resolution mode (resolution 20000 FWHM). The optimized conditions in negative mode for the sample included capillary voltage; 2 kV, sample cone voltage; 40 V, source temperature; 80 °C, desolvation temperature, 250 °C; desolvation gas flow (N_2_), 1200 L·h^−1^. For MS and MS/MS experiments, data were acquired in the 50–2500 *m/z* range with 1s scan time and 0.02 s interscan delay. Sample solutions of 17 μM duplex or dendriplex were infused into the source at a flow-rate of 3 µL·min^−1^ by means of a syringe pump (Cole-Palmer, Vernon Hills, IL, USA).

*ESI-Q-TOF-MS/MS*: These experiments involved: (i) selection of the [M−5H]^5−^ precursor ion with the quadrupole mass analyzer (ii) fragmentation in the trap collision cell (first ion guide of the Triwave, collision voltage; 20 V–40 V).

IMS conditions were optimized for each dendriplex samples as follows:

[*ds*/φ3G0(N)] dendriplex solution: gas flow (N_2_): 80 mL·min^−1^, wave height: 30 V, and wave velocities: 1200 m·s^−1^.

[*ds*/Phe_n_G1(N)] dendriplex solution: gas flow (N_2_): 100 mL·min^−1^, wave height: 40 V, and wave velocities: 1200 m·s^−1^.

### 3.5. ESI-QIT-MS(/MS) of Dendriplexes [ds/Native PAMAM] and [ds/Modified- PAMAM] 

*ESI-QIT-MS(/MS)*: These experiments were performed using a Bruker HCT Ultra ETD II quadrupole ion trap (QIT) mass spectrometer equipped with an ESI source and the Esquire control 6.2 and Data Analysis 4.0 software (Bruker Daltonics, Bremen, Germany). For the ESI parameters the capillary and end plate offset voltages were respectively set to 2.0 kV and −0.5 kV in negative mode. The skimmer voltage was set to −40 V and the injection low mass cut off (LMCO, corresponding to the “trap drive” parameter) value was *m/z* 128. The nebulizer gas (N_2_) pressure, drying gas (N_2_) flow rate and drying gas temperature were 7 psi, 5.0 L·min^−1^ and 250 °C, respectively. Helium pressure in the ion trap was 9.6 × 10^−6^ mbar. Spectra were acquired in the *m/z* 100–3000 range, using the scan rate of 8000 *m/z* units per second (‘Standard-Enhanced’ mode). The number of ions entering the ion trap was automatically adjusted by controlling the accumulation time with the ion charge control (ICC) mode (target 200000) with a maximum accumulation time of 50 ms. The values of spectra averages and rolling average were 10 and 5 respectively. ESI-MS/MS experiments were carried out by collision-induced dissociation (CID) using a resonant excitation with amplitudes from 0.6 to 1.0 V_p-p_, helium as the collision gas, isolation width of 1 *m/z* unit for the precursor ions. Sample solutions of 17 μM of duplex or dendriplex were infused into the source at a flow-rate of 3 µL·min^−1^ by means of a syringe pump (Cole-Palmer).

## 4. Conclusions

This work has reported the first study of [DNA duplex/PAMAM] dendriplexes by ion mobility-mass spectrometry. The resort to the IM-MS bidimensional technique for the study of dendriplexes permitted to evidence species that could not be distinguished by mass spectrometry only. The unambiguous identification of the different species present in the samples; expected dendriplexes, possible additional non-specific complexes and free ligands is possible since the problem of MS signal overlapping is solved by ion separations in the ion mobility cell. We demonstrated that the fully modified PAMAM (φ_3_G0(N)) and the native PAMAM G0(N) have the same affinity for DNA duplex d(GCGCAATTGCGC)_2_ (K_D_ in the 10^−4^ M range). However, different binding modes could be envisaged for these ligands; electrostatic interactions were expected in the case of the [*ds*/G0(N)] dendriplex while the presence of the phenyl groups in (φ_3_G0(N) allowed to envisage intercalation between base pairs of DNA. Indeed, different behaviors were observed for the two dendriplexes; (i) in positive-ion ESI-MS the ions of [*ds*/G0(N)] dendriplex could be detected but not the ions of [*ds*/φ_3_G0(N)] dendriplex; (ii) the dissociation of [*ds*/G0(N)-5H]^5−^ ion yielded complementary product ions [*ss*/G0(N)-2H]^2−^ and ([*ss*-3H]^3−^) arising from strand separation while the dissociation of [*ds*/φ_3_G0(N)/5H]^5−^ led to the complementary product ions of modified PAMAM ([φ_3_G0(N)-H]^−^) and DNA duplex ([ds-4H]^4−^). The larger PAMAM G1(N) was partially modified with phenylalanine to both preserve the cationic property and add hydrophobic groups. However, the affinities of native and phenylalanine modified G1(N) PAMAM for the DNA duplex were almost similar and very low; indeed, the corresponding dendriplex ions were of very low relative abundance. These weak affinities do not favor the formation of strong host-guest complexes but could constitute an advantageous issue for gene delivery according to [2]. Future perspectives of this work can consist in charge:ratio investigations, ITC or fluorescence based-experiments for binding constants determination and study of other PAMAM systems (other chemical derivation, higher generation…) and also other DNA systems. Moreover, because *in vivo* delivery of dendrimer-DNA complex is expected to encounter pHs over a range of 5.0–7.4, the study of complexation with pH will also constitute another perspective.

## Supplementary Materials

Supplementary materials can be accessed at: http://www.mdpi.com/1420-3049/19/12/20731/s1.
