# Supplementary material for: Investigation of Dendriplexes by Ion Mobility-Mass Spectrometry
Source: Molecules. 2014 Dec 12;19(12):20731–50. doi: 10.3390/molecules191220731 (PMC6271531; doi:10.3390/molecules191220731)

# Supplementary Materials

**Figure S1.** Carbon labelling of (**A**) $\phi_3$G0(N), (**B**) G0.5(N) and (**C**) Phe$_3$G1(N).

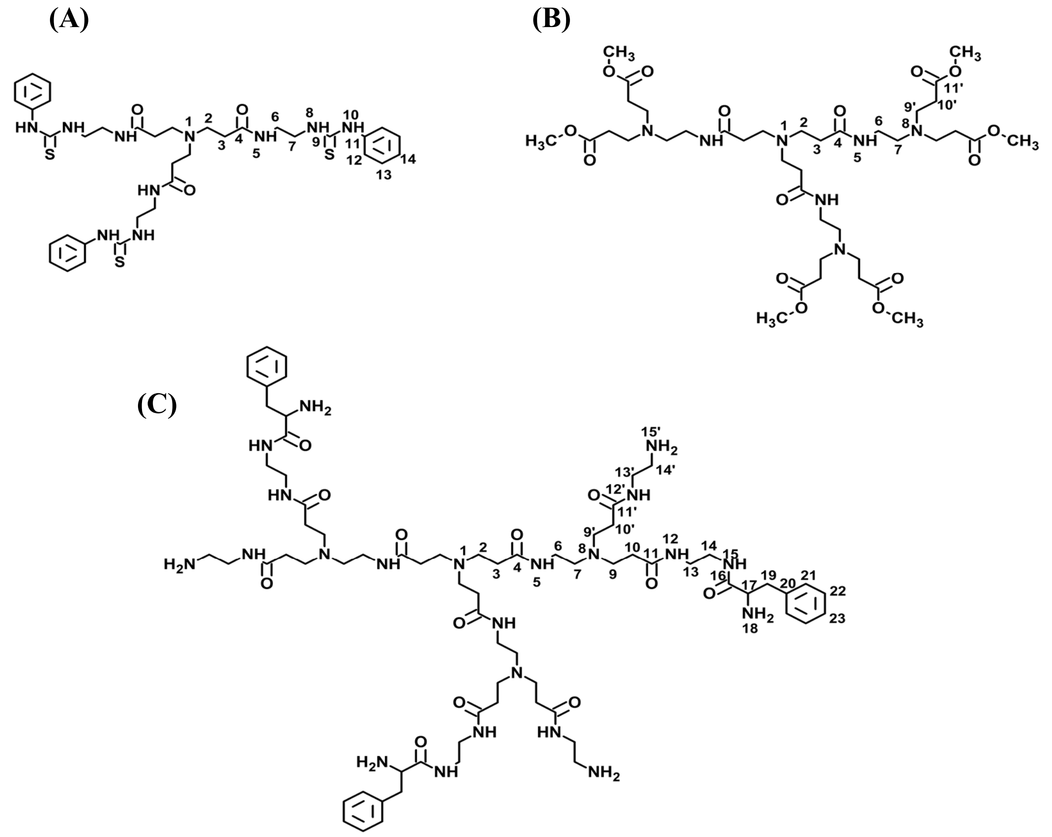


**Figure S2.** Positive ion ESI-MS/MS mass spectrum of $[\phi_3G0(N)+H]^+$ and the corresponding fragmentation pattern.

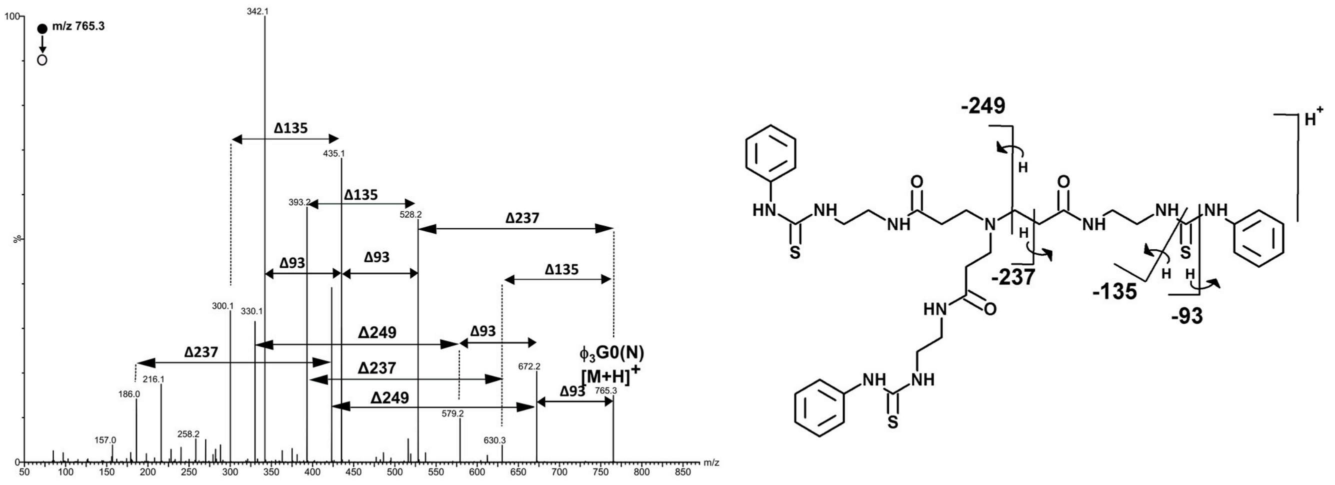

**Figure S3.** ESI-MS/MS mass spectrum Phe2G1(N) (**A**) and the corresponding fragmentation pattern (**B**).

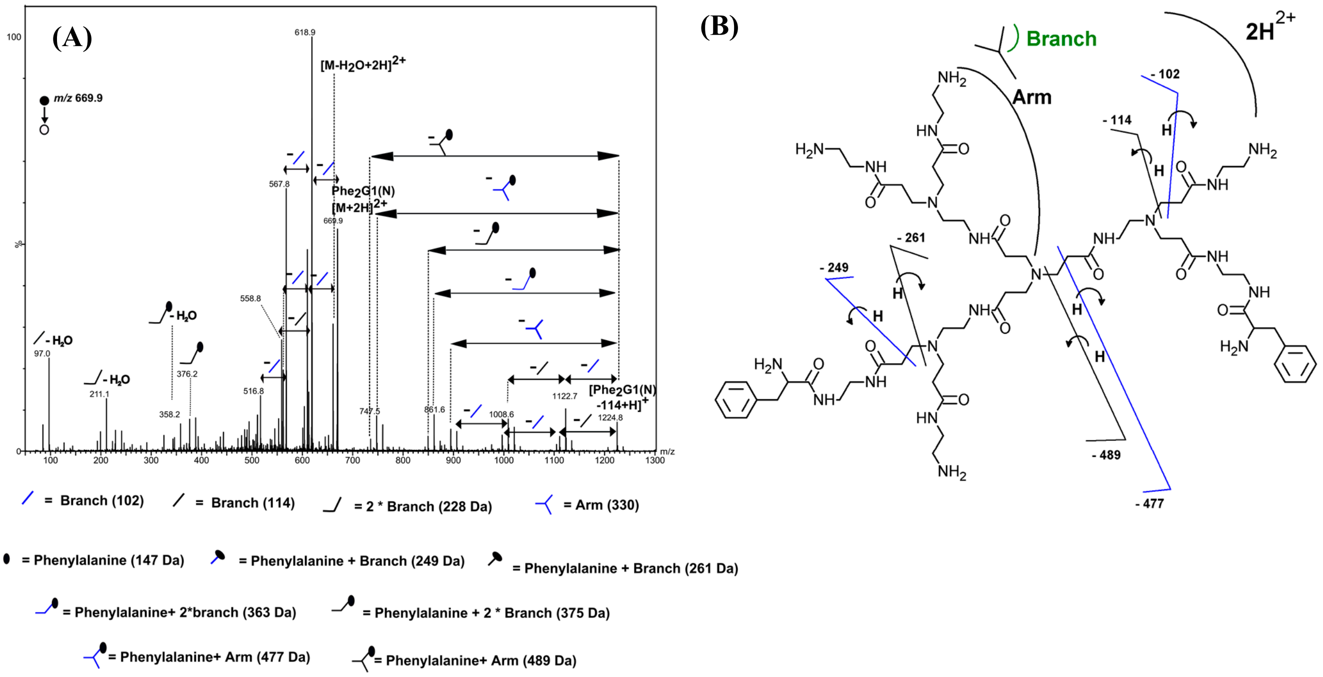

Supplement: Supplementary file 1 [file molecules-19-20731-s001.pdf]
